# Global Delivery of Foetal Sequencing: Do We Need Some Standardisation?

**DOI:** 10.1002/pd.6866

**Published:** 2025-07-25

**Authors:** Natalie J. Chandler, Zandra C. Deans

**Affiliations:** ^1^ NHS North Thames Genomic Laboratory Hub Great Ormond Street Hospital for Children NHS Foundation Trust London UK; ^2^ Genetics and Genomic Medicine UCL Great Ormond Street Institute of Child Health London UK; ^3^ GenQA Laboratory Medicine Royal Infirmary of Edinburgh Edinburgh UK

## Abstract

**Objective:**

The development of sequencing technologies has resulted in rapid expansion in the testing available for foetuses with structural anomalies to diagnose monogenic disorders. To understand the variability in how foetal sequencing services are delivered, we developed a survey that focussed on the scope of testing, any parallel testing performed, laboratory and analytical processes, multidisciplinary team working, reporting practices, quality, reanalysis and data sharing.

**Method:**

A draft survey was developed and reviewed by members of the International Society of Prenatal Diagnosis (ISPD) and revised accordingly. Questions were developed with the aim of ascertaining how prenatal sequencing services are being conducted and results reported. The survey was distributed to members of all GenQA registered laboratories and ISPD members.

**Results:**

Responses were received from 101 individuals from a range of specialisms. The results show a high degree of variability in how laboratories are conducting, analysing and reporting foetal sequencing tests.

**Conclusion:**

The survey results demonstrate the need for global guidance on issues related specifically to prenatal sequencing. To include: the role of the clinical team prior to testing, the scope and limitations of sequencing, multidisciplinary working to interpret the data, the handling unexpected findings and clear, accurate reporting of the results.

## Summary

- What's already known about this topic?
  ◦ Many laboratories provide a clinical foetal sequencing service, but there is currently no universal standardisation or guidance to ensure high‐quality testing and reporting.
- What does this study add?
  ◦ A detailed review of the global delivery of foetal sequencing has identified some areas of good practice, including the use of variant classification guidelines and not solely relying on inheritance filtering with trio sequencing. However, there are others, such as accreditation, external quality assessment, reporting and multidisciplinary team working where standardisation is required to ensure safe and ethical patient care.

## 1 Introduction

The development of sequencing technologies has resulted in a rapid expansion in the testing available to diagnose monogenic disorders in foetuses with structural anomalies, with a systematic review determining that sequencing has an incremental diagnostic yield of 31% in foetuses with non‐diagnostic chromosomal microarray or karyotype [1]. There are numerous research studies investigating indications for foetal sequencing and diagnostic yield for each indication, however studies investigating the laboratory's involvement from testing and bioinformatic analytical strategies to reporting and participation in multidisciplinary team (MDT) discussions are lacking.

In general, foetal sequencing strategies have been referred to as exome sequencing and have been focussed primarily on the detection of single nucleotide variant (SNVs) and small insertion deletions [1, 2]. However, the Mellis et al. systematic review found other approaches are in use—18/72 studies used clinical exome (capture of all known disease‐causing gene) sequencing and 4/72 used genome sequencing. Recently, there have been several more publications using genome sequencing and analysis strategies are rapidly being developed to detect all types of sequencing variants. However, targeted panels remain in use in several laboratories [3, 4]. There are only a few studies that compare these approaches [4, 5]. Analytical approaches also vary with some studies using panel approaches to filter variants for analysis [6], some using agnostic approaches with inheritance filtering [7], phenotype filtering [8] or commercial solutions that consider both [4]. Indeed, the Mellis et al. systematic review shows that 37% of prenatal sequencing studies provide only partial or no information regarding the variant filtering and prioritisation strategy and this only covered SNVs.

Reporting strategy also shows variation with some international societies recommending that no variants of uncertain significance (VUS) are reported [2], some that VUS are reported if they occur in genes that strongly fit the foetal phenotype [9], some that VUS are reported if agreed at MDT [10] and other studies showing that some laboratories are reporting more than one VUS in 37% of patients [3]. There is also no international consensus on reporting incidental or additional findings, resulting in variability in practice [2]. MDT working is recommended in international statements [2, 9] and has been shown in research studies to improve outcomes [11, 12] however, there is scarce information in sequencing studies detailing if and when these occur in the testing pathway and who is included.

These reports demonstrate that laboratory testing, analytical and reporting practices vary across the globe and depend upon demand, funding, capability and capacity of clinical and laboratory services. However, it is challenging to determine the variation as there is limited information included in reports of foetal sequencing [1]. Without a clear understanding as to how these services are being delivered, it is difficult to compare services or identify areas of best practice that could form the basis of recommendations to standardisation of approaches and level of service. One mechanism of identifying the variation is through mining the data derived from external quality assessment (EQA) schemes or utilising the engagement of EQA participating laboratories and membership of professional bodies giving insight into real world activity. To build a picture of the variety in how foetal sequencing services are being conducted, we developed a survey that focussed on the scope of testing, parallel testing being performed, laboratory and analytical processes for both SNVs and CNVs, MDT working, reporting practices, quality, reanalysis and data sharing.

## 2 Methods

### 2.1 Study Design

A draft survey was developed and reviewed by ISPD members with experience of foetal sequencing and was revised accordingly. The questions were developed with the aim of ascertaining information regarding how prenatal sequencing services are being conducted and reported by laboratories. The scope of the survey covered the role of the respondent, current local foetal sequencing provision, testing strategies, parallel testing options, turnaround times, data analysis, processes to handle incidental and additional looked‐for findings, reporting formats, EQA participation and accreditation status. The survey questions were created in word (see Supporting Information S1: supplementary data) and the online survey was administered via survey monkey (SurveyMonkey—www.surveymonkey.com). The survey was designed so that it was not compulsory to answer every question as we anticipated not all staff groups would be able to answer all of the questions, for example detailed technical questions might only be understood by scientists and not clinicians.

### 2.2 Distribution of Survey

The survey was distributed to all GenQA registered laboratories and ISPD members. An introductory email was sent in September 2022 and the survey was live during September through December 2022 then again 1st February 2023 to 31st March 2023.

### 2.3 Data Analysis

Data were extracted and analysed using Excel (Microsoft Corporation, Redmond, WA). As the survey was designed so that not all questions were mandatory, there was variation in the number of responses for each question. All results are therefore given as a percentage of the responses received for that question rather than the total number of respondents to the survey.

## 3 Results

### 3.1 Survey Respondent Characteristics

**FIGURE 1:**
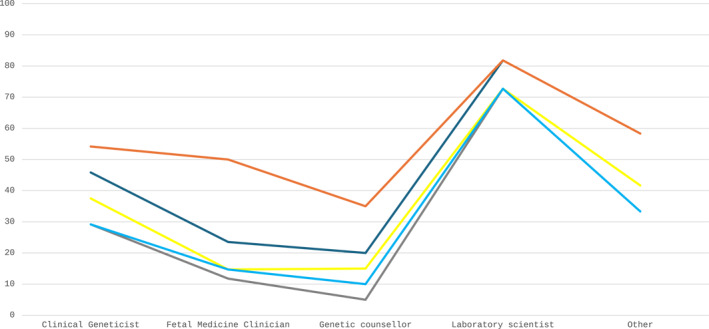
The percentage of respondents from each self‐reported role that answered the following questions. What is captured (orange)? What is analysed (dark blue)? Is the service accredited (yellow)? Does laboratory participate in EQA (light blue)? What variant strategies are used (grey)?

Responses were received from 101 individuals from 20 countries covering five continents. A list of the responses (*n* = 101) by country and the percentage from each country is in Supporting Information S1: supplementary table 1. 88.1% of the responses were from high income (according to the world bank classification) countries with 53.9% of responses from laboratories that perform prenatal sequencing. Twelve responses were received from six lower‐to middle‐income countries with 75% performing prenatal sequencing. In relation to the roles of respondents, there were 23.8% (*n* = 24) Clinical Geneticists, 33.7% (*n* = 34) Foetal Medicine Clinicians, 19.8% (*n* = 20) Genetic counsellors, 10.9% (*n* = 11) Laboratory Scientists and 11.9% (*n* = 12) others, which comprised of a combination of trainees and industry employees and combinations of all other roles. To gauge an understanding of the knowledge of each role regarding different laboratory processes, a subset of four questions were selected to look at the percentage response rate: (1) what is captured? (2) what is analysed? (3) is the service accredited? (4) Does the laboratory participate in EQA (Figure 1)? The results show response rates from highest to lowest for the following roles: Laboratory scientists, Other, Clinical Geneticists, Foetal Medicine Clinicians, Genetic counsellors. The pattern was the same for all four questions.

### 3.2 Scope of Testing

Foetal sequencing was performed by approximately 60 (yes):40 (no) split (total responses = 100) and those who indicated it was being implemented (*n* = 15), five gave timelines up to 2025 with a further four by 2030. Of the 49 responses from centres performing foetal sequencing, the majority of services had tested < 50 probands in the previous 12 months (47%), 16.3% tested between 51 and 100, 18.4% 101–200, 14.3% 201%–500% and 4.1% testing > 500 probands. Respondents were asked for the target reporting turnaround times from receipt of sample(s) to issuing the report‐from the 49 responses, over 60% delivered results within 1–3 weeks (22.4% 1–2 weeks; 38.9% 2–3 weeks), 30.6% reported results between 3–4 weeks and small numbers were outliers providing results either within one week (2%) or longer than one month (6.1%). Multiple sample types were tested by laboratories (responses *n* = 55), with all accepting amniotic fluid and 92.7% accepting chorionic villus samples. Foetal blood, cultured cells and DNA were accepted by 65.5%, 67.3% and 65.5%, respectively, and 10.9% would test foetal tissue samples. The bulk of the responders (*n* = 53) preferred to receive trio family structures (94.3%), 3.8% were content to test singletons and 1.9% would accept samples from a range of different family members. In reality, 90.6% tested trios, 47.2% duos and 54.7% singletons.

### 3.3 Parallel Testing

Common aneuploidy testing was performed as well as sequencing by 90.5% of respondents (*n* = 63). From the 54 responses, this was carried out prior to referral for sequencing by 50.0% and in parallel by 24.0% with the remaining stating it would be performed either prior or in parallel depending on gestational age and/or ultrasound findings and/or whether counselling had taken place.

The majority of the 62 responses also performed microarray analysis (83.9%) with 8.1% not performing microarray analysis and 8.1% sometimes performing microarray (dependent upon patient choice, whether consent taken prior to sequencing and if exome sequencing service is able to detect copy number variants (CNVs)). Of the 49 responses, microarrays were performed prior to foetal sequencing by 42.9% of services or in parallel by 30.6%. The remaining outlined multiple approaches.

Prior to sequencing, 76.8% of the total responses (*n* = 56) test for maternal cell contamination in the foetal sample with 63.9% proceeding if no ‘significant contamination’ is detected. This is defined as < 10% by a further 13.9%. The remaining respondents stated either it was not known or testing was performed by remote services and the approach varied.

### 3.4 Library Preparation and Analytical Strategies

**FIGURE 2:**
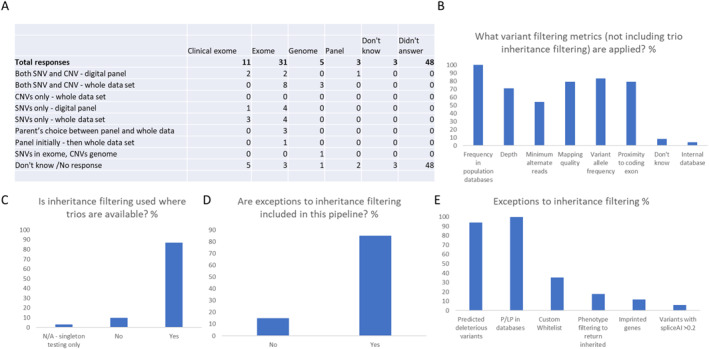
Library preparation and analytical strategies. The library capture used and region included in the analysis pipeline (A). The variant filtering metrics applied to filter variants (B). The responses to the question ‘Is inheritance filtering used where trios are available’? (C), whether exceptions to inheritance filtering are applied (D) and what these exceptions are (E).

Of the respondents that answered (*n* = 53) or did not select ‘don't know’, exome capture (62.0%) was the most common library preparation method, followed by Clinical Exome (definition given to survey respondents: capture of all known disease‐causing genes) (22.0%), genome (10.0%) and panels (6.0%) (Figure 2A). There was a large variation in the responses (*n* = 39) to the ‘what is analysed’ question. For genome sequencing, wider approaches are used rather than phenotypic/foetal anomaly panels. The most common response for exome sequencing was analysing the whole data set for both SNVs and CNVs, but there were also responses indicating that parent's choose which analytical approach to take. The most common variant filtering strategy, from 24 respondents, is frequency in population databases, but other quality metrics such as depth, variant allele frequency, and mapping quality are also commonly used (Figure 2B). 87.1% of respondents (*n* = 31) use inheritance patterns to filter variants (Figure 2C) with 15% of respondents solely relying on this strategy (Figure 2D). The remaining 85% include exceptions, which are shown in Figure 2E. Notably, only 17.6% of respondents indicated that they used phenotypic filtering to return inherited variants if they fit the foetal phenotype.

### 3.5 Variant Classification and Confirmation

**FIGURE 3:**
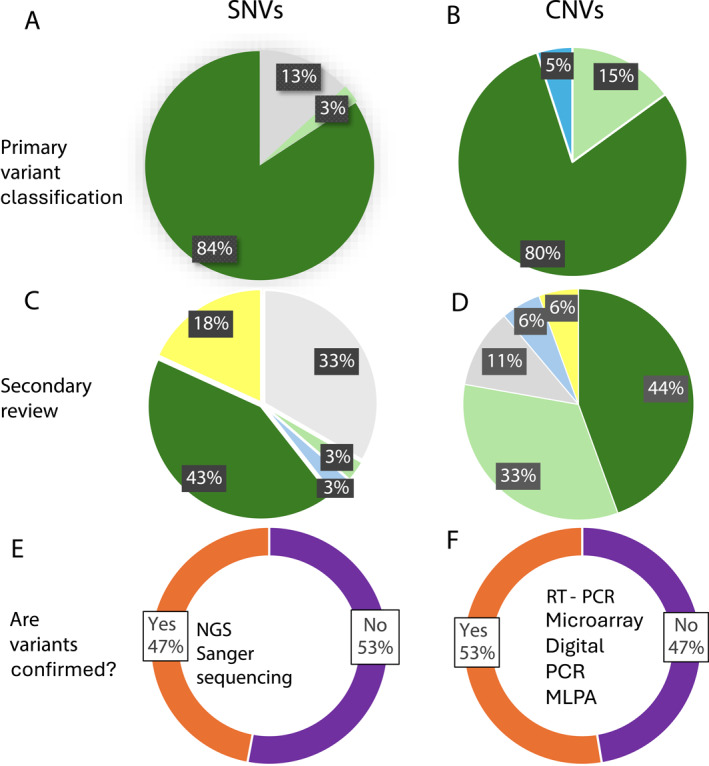
Variant classification. The percentage of self‐reported staff roles that carry out the primary variant classification for SNVs (A) and CNVs (B). The percentage of self‐reported staff roles that carry out the secondary review of the variant classification for SNVs (C) and CNVs (D) (green = laboratory team; Grey = Clinical Geneticist; light green = Genetic counsellor; yellow = MDT review; blue = foetal medicine clinician). Reponses to the question ‘Are variants confirmed’? for SNVs (E) and CNVs (F) with the techniques listed in the centre.

Figure 3 shows the responses for who performs variant classification of SNVs (*n* = 38) (Figure 3A and 3C) and CNVs (*n* = 20) (Figure 3B and 3D) and whether they are confirmed (Figure 3E and 3F). Of interest, only 67.6% of responses (*n* = 34) indicated that CNVs are analysed from sequencing data. For SNVs 82.5% are classified according to the American college of Medical Genetics (ACMG) criteria [13] and 12.5% according to the United Kingdom Association of Clinical Genomic Science (ACGS) criteria [14] with one respondent using their own guidelines based on ACMG guidelines and another working on implementation of these guidelines. 94.3% of respondents had at least two reviews of the SNV variant classification. In 83.6% of cases, the first review was performed by the laboratory team, but this was only 41.1% for the secondary review. Secondary review was performed by Clinical Geneticists (32.4%), foetal medicine clinicians (2.9%), genetic counsellors (2.9%) and MDT review (17.6%). CNVs are analysed from sequencing data by 67.6% of respondents with 68% classifying according to ACMG CNV guidelines [15], 13.6% according to ACGS guidelines [14] and four other responses (neither guidelines are a good fit for all CNVs; unsure, national guidelines, own guidelines based on ACMG). All respondents perform at least two reviews of CNVs with the initial review being performed by the laboratory team in 80% of cases, whereby it is only 44.4% for the secondary review. Secondary review was performed by Clinical Geneticists (33.3%), foetal medicine clinical (5.6%), genetic counsellors (11.1%) and MDT review (5.6%)

### 3.6 Multidisciplinary Team Working

**FIGURE 4:**
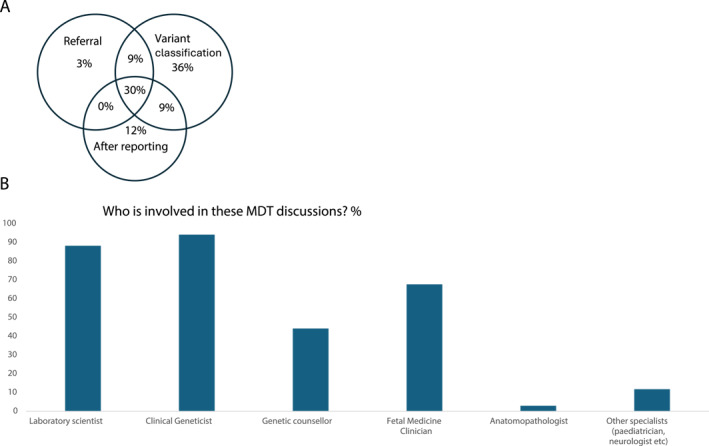
Multidisciplinary team (MDT) working. (A) Venn diagram showing at what stages in the testing process MDT discussions are helpful. (B) The roles of staff involved in MDT discussions.

Figure 4A shows the different points in the testing pathway where MDT meetings occur where there were 34 responses. These vary and include the point of referral and discussion of variant classification prior to reporting or after reporting with some respondents indicating that they occur at multiple points. The majority of MDT meetings were held prior to reporting with only 12% holding these solely after reporting. Two respondents indicated that they did not conduct MDT discussions for all cases but were available on a case‐by‐case basis. Figure 4B shows the specialities of those attending the MDT meetings.

### 3.7 Reporting

All respondents (*n* = 33) agreed that the report should contain a brief description of the bioinformatics pipeline, variant prioritisation and classification protocols employed, if the result is consistent with/confirms a diagnosis or not, the mode of inheritance of the variant (if known), and whether testing of other family members and prenatal testing for future pregnancies should be offered. With regard to how the variant is reported, 97% recommended using the HGVS nomenclature [16] and 88% wished to see the criteria used to classify the pathogenicity of the variant. Approximately half of the responders (48%) also wanted the outcome of the MDT to be stated for example, a recommendation for the postnatal phenotypic outcome to be followed up, but only one individual required the available treatments to be included in the report. For the 27 responses to the question regarding ‘what technical information is covered on the report’, details of the genes analysed in the form of gene panel version or link to the gene list was required by 81% and the mean coverage or link to determine the coverage of specific genes was a requirement by 77% of individuals. There was less interest in knowing the validated sensitivity of the bioinformatics pipeline (48%) or the limitations of the test performed (4%). Of 23 total responses, the majority of individuals shared variant data (70%), predominantly using ClinVar (45%), then Decipher (33%), VKGL‐database in the Netherlands (12.5%), Franklin (4%) and the remaining intending to submit data in the future.

### 3.8 Quality

Out of 30 responses, the majority of laboratories (73%) are accredited for the service with 70% being accredited to ISO:15189. Equal numbers of laboratories were accredited to/by the following alternatives: ISO17025, College of American Pathologists, Israeli Ministry of Health, National Association of Testing Authorities (NATA), or the accreditation standard/body was not known. Approximately half of the 26 respondents (54%) participated in external quality assessment (EQA) or proficiency testing (PT), the majority of which used the services of GenQA (64%), EMQN (22%), CAP (7%) and 7% performed a data swap exercise with a third party.

### 3.9 Findings Not Linked to Reason for Testing

**FIGURE 5:**
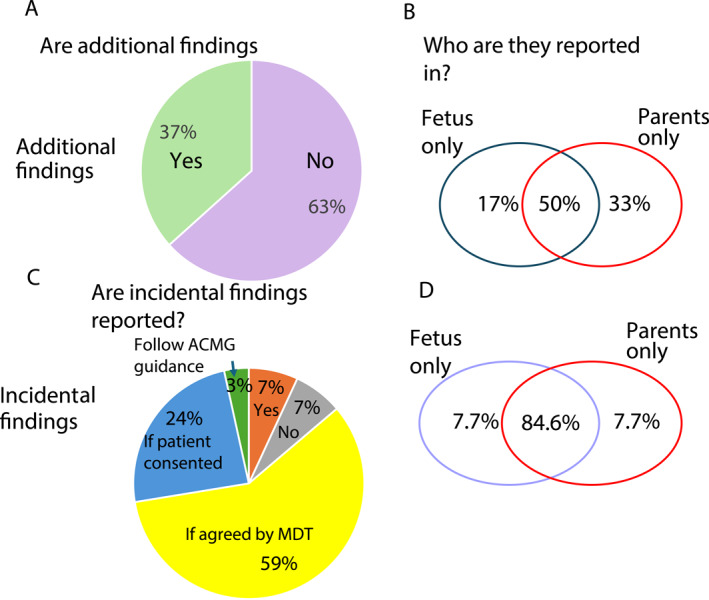
Additional and incidental findings. (A) Responses to the question ‘Are additional findings looked for’? light green = Yes; purple = No. (B) Responses to ‘Who are additional findings reported in’? (C) Responses to the question ‘Are incidental findings reported’? Yellow = yes—if agreed by MDT; Blue = Other—depends on what patients have consented for; Orange = yes always, Grey = No, Green = Other—the laboratory follow ACMG guidance. (D) Responses to ‘Who are incidental findings reported in’?

There were 30 respondents for the questions regarding additional findings, which are not linked to the reason for testing the foetus but are specifically looked for and analysed. These were reported by 36.7% of services (Figure 5A). The majority of these (66.7%) complied with the ACMG gene list [17] with the remaining adding additional genes for example, those related to metabolic conditions, intellectual disability syndromes, or those relevant to the family as indicated by the clinical team. These findings were reported whether they were detected in the foetus or the parents by half of the laboratories; one third reported the results for the parents only and the remainder for the foetus only (Figure 5B).

For incidental findings, where pertinent genomic results are detected which are not specifically looked for or not related to the foetal findings, there were 29 responses. Incidental findings were automatically reported by only 6.9% of survey respondents. Many indicated they preferred to discuss at MDT meetings before deciding whether to report or not, and 6.9% stated incidental findings would never be reported (Figure 5C). When incidental findings were reported, 84.6% would report for all family members and the remaining would equally report only for the parents or for the foetus (Figure 5D).

### 3.10 Reanalysis

There were 27 responses to the questions regarding reanalysis. Reanalysis of the data is performed in 81.5% of cases in response to an evolving phenotype post birth, a new pregnancy in the family, an updated virtual gene panel, or reanalysis is systematic/planned.

## 4 Discussion

There was a wide range of responses from different countries and variable roles in the workforce giving an indication of the differing approaches employed for a clinical foetal sequencing service. One limitation, however, is that some staff groups may have tried to answer some questions out of their area of expertise, so inaccuracies may be present in the data. The membership of ISPD and GenQA encompasses multiple genomic and clinical services and so, inevitably, the survey was sent to some individuals with no involvement in foetal sequencing. Another limitation was that the survey was only available in English; therefore, this may have impacted the response rate and understanding of the questions asked.

The testing and variant review strategies and technical details were well understood by the Scientists completing the survey. However, in general, the responses from Clinicians indicated inadequate understanding of the scope of the test being performed, that is, what is analysed and reported and what is not, the impact of different bioinformatic pipelines on the results obtained, the potential for reporting incidental findings, amongst other issues. These are important facts and clinicians need to be aware of details to ensure accurate counselling of parents both before testing and when discussing results. It is important that there is close working between the clinical service and the laboratory teams to ensure that all involved are aware of the service being delivered, whether through personal interaction, MDT working, or information included on the clinical reports.

As foetal phenotyping is challenging, the interpretation of genomic data in the context of the foetal anomalies can be difficult. Again, this is where discussion of the findings with the Clinicians who have seen the family is critical in determining if the variant is the cause of the phenotype and whether further investigation may be required. This is particularly pertinent as prenatal phenotypes can evolve [6, 18]. Furthermore, the sharing of genomic data alongside the associated phenotypes, including post‐natal features where available, can support diagnoses and reduce the burden and time required to reach a genomic conclusion in future cases. It is recognised that obtaining local and often national permission to share data and comply with the requirements for information governance is difficult and the genomic community must work globally to ensure this becomes second nature to maximise the benefits of genomic medicine for all.

Some variation in practice across the globe is inevitable as laws, local availability and other factors will vary across jurisdictions; however, in Table 1 we suggest recommendations for best practice in light of the outcomes from this study. However, once good practice has been established, it is important to ensure implementation and monitoring of compliance. This is addressed partially through accreditation of the foetal sequencing service however the nuances specific to the service are best measured through regular participation in EQA which provides not only proficiency testing that is demonstration that the correct result was obtained, and benchmarking of the service against peers, but also an educational element to identify areas of suboptimal working. EQA providers can enable the provision of expert advice to support improvement in the service and in essence drive standardisation and service development through best practice.

**TABLE 1:** Recommendations for delivering a foetal sequencing service following findings from the survey.

Scope	Recommendation	Benefit
Service provided	The clinical team(s) must be aware of the service provided with associated limitations to enable appropriate pretest counselling.	The family is aware prior to testing of the scope of the test and the associated limitations.
Interpretation of results	Findings should be discussed between scientific and clinical teams to identify variants pertinent to the case.	Ensure appropriate association of variants with phenotype. Increase diagnostic yield and ensure evolving phenotypic information is used to aid data interpretation.
Reporting of results	The report should clearly state what testing has been performed, the result and whether the findings relate to the phenotype.	Clear reporting of the result to minimise misinterpretation.
Quality	Laboratories should participate in external quality assessment and gain ISO 15189 or equivalent accreditation.	Ensure quality of service and identify and implement service development.
Data sharing	Variant and phenotypic data should be shared nationally and internationally through variant databases and publication to aid future diagnosis.	Aid interpretation of the data for all and improve time to diagnosis.

## 5 Conclusions

As with the development and implementation of any new clinical genomic service, there is a lack of standardisation and agreed best practice for foetal sequencing. As services have evolved over the past few years, there is now a better understanding of the challenges encountered to deliver safe, accurate and acceptable testing and reporting. Although guidelines do exist [9], our survey results demonstrate there is still variability and the need for updated global guidance on issues related specifically to prenatal sequencing, which need to encompass cytogenomic variants as well as SNVs. A complexity in counselling for foetal sequencing, unlike other technologies used prenatally, is the routine testing of parental samples, which has further implications for the identification of incidental and additional findings. Global recommendations need to address the role of the clinical team prior to testing, the scope and limitations of the sequencing, multidisciplinary working to interpret the data, the handling of unexpected findings and clear, accurate reporting of the results. Our results show that there is an educational gap, particularly amongst clinicians, who need to understand the limitations of the test being offered to enable accurate pre‐ and post‐test counselling. This may best be delivered by more MDT working.

## Ethics Statement

The study followed the principles of the Declaration of Helsinki [19]. Ethical approval by the NHS Research Ethics Committee (REC) review for England was deemed not necessary as the study participants were registered healthcare professionals. Further research and development approval was confirmed not to be needed.

## Consent

Information on the scope of the survey was provided to all and consent was obtained for all survey respondents prior to the completion of the survey.

## Conflicts of Interest

The authors declare no conflicts of interest.

## Supporting information

Supporting Information S1

## Data Availability

The data that support the findings of this study are available on reasonable request from the corresponding author. The data are not publicly available due to privacy or ethical restrictions.
