# Supplementary material for: Global Delivery of Foetal Sequencing: Do We Need Some Standardisation?
Source: Prenat Diagn. 2025 Jul 25;46(5-6):650–8. doi: 10.1002/pd.6866 (PMC13170051; doi:10.1002/pd.6866)
Supplement: Supplementary file 1 — Supporting Information S1 [file PD-46-650-s001.docx]

Supplementary table 1 – the number of responses by country and percentage from each country already performing prenatal sequencing when the survey was conducted. The countries in black cells with white text are lower- and middle-income countries according to the World Bank.

| Country | Number of responses | % laboratory performs prenatal sequencing |
| --- | --- | --- |
| Australia | 4 | 100% |
| Belgium | 3 | 100% |
| Bulgaria | 1 | 0% |
| Canada | 13 | 7.7% |
| China | 4 | 100% |
| Denmark | 1 | 100% |
| Ethiopia | 1 | 0% |
| France | 2 | 100% |
| Germany | 3 | 66.7% |
| Greece | 1 | 100% |
| Hong Kong | 2 | 50% |
| India | 4 | 75% |
| Indonesia | 1 | 0% |
| Ireland | 1 | 0% |
| Israel | 1 | 100% |
| Japan | 2 | 50% |
| Netherlands | 8 | 100% |
| Portugal | 1 | 0% |
| Saudi Arabia | 1 | 100% |
| Singapore | 2 | 0% |
| Spain | 2 | 100% |
| Switzerland | 2 | 100% |
| United Arab Emirates | 2 | 100% |
| United Kingdom | 6 | 66.7% |
| United States | 31 | 41.9% |
| Vietnam | 2 | 100% |

Supplementary data – the complete survey content:

| **General Information** | | | | | | | |
| --- | --- | --- | --- | --- | --- | --- | --- |
| Please indicate the role you play in the prenatal exome sequencing service. | | - Laboratory scientist - Clinical Geneticist - Genetic counsellor - Fetal Medicine Clinician - Other – please state | | | | | |
| Please enter your country | | - Dropdown list of countries | | | | | |
| **Service** | | | | | | | |
| Does your laboratory perform clinical prenatal exome / genome sequencing? | No | | Does your laboratory plan to implement clinical prenatal exome / genome sequencing? | No | Move to end of survey and thank you page |  |  |
|  |  |  |  | Yes | When do you expect to go-live with clinical testing? | Year dropdown menu | Move to next section |
|  | Yes | | When did you start testing? | Year dropdown menu | Move to next question |  |  |
|  |  | | How many cases i.e. probands tested in the last 12 months? | <50  51-100  101-200  201-500  >500 | Move to next question |  |  |
|  |  | | What is your target reporting turnaround time from receipt of samples? | <1 week  1-2weeks  2-3weeks  3 weeks – 1month  >1 month | Move to next section |  |  |
| Pre-testing / parallel testing | | | | | | | |
| Is common aneuploidy testing performed? | Yes | | When is this testing performed? | - Prior to referral - In parallel - Other – please state | Move to next question |  |  |
|  | No | | Move to next question |  |  |  |  |
| Is microarray testing performed? | Yes | | When is this testing performed? | - Prior to referral - In parallel - Other – please state | Move to next question |  |  |
|  | No | | Move to next question |  |  |  |  |
|  | Sometimes – please state | | When is this testing performed? | - Prior to referral - In parallel   Other – please state | Move to next question |  |  |
| Is maternal cell contamination assessed prior to testing | Yes | | What is the maternal cell contamination cut off for testing to be performed? | - No significant MCC only - 10% - 20% - 30 - 40% - Other (please state) | Move to next section |  |  |
|  | No | | Move to next section |  |  |  |  |
| Laboratory processing and techniques | | | | | | | |
| What types of sample are accepted for testing? (select all that apply) | - Chorionic villus sampling - Amniotic fluid - Fetal blood - Cultured cells - DNA - Other – please state | | Move to next Question |  |  |  |  |
| What family structures are tested? (select all that apply) | - Trio - Singleton - Duo - Other – please state | | Which is your preferred family structure for testing? | - Trio - Singleton - Duo - Other – please state | Move to next question |  |  |
| Type of sequencing capture performed | - Genome - Exome - Clinical exome (capture of all known disease-causing genes) - Panel - Other (please specify) | | If panel selected: | Please provide details of panel (weblink or free typing list of genes) | Move to next section |  |  |
| Variant filtering – SNVs (single nucleotide variants) and CNVs (copy number variants) | | | | | | | |
| What types of variants do you analyse and do you analyse the whole dataset or apply a digital panel (i.e. a panel of genes is applied during the bioinformatic filtering)? | SNVs only - whole data set | | Move to next question |  |  |  |  |
|  | Both SNVs and CNVs - whole data set | | Move to next question |  |  |  |  |
|  | CNVs only - whole data set | | Move to next question |  |  |  |  |
|  | SNVs only - Digital panel | | Please provide details of panel (weblink or free typing list of genes) | Move to next question |  |  |  |
|  | Both SNVs and CNVs - Digital panel | | Please provide details of panel (weblink or free typing list of genes) | Move to next question |  |  |  |
|  | CNVs only - Digital panel | | Please provide details of panel (weblink or free typing list of genes) | Move to next question |  |  |  |
|  | Other – please specify | |  | Move to next question |  |  |  |
| What variant filtering metrics (not including trio inheritance filtering) are applied? – tick all that apply | - Frequency in population databases e.g. gnomAD - Depth - Minimum alternate reads - Mapping quality - Variant allele frequency - Proximity to coding exon - Other – please state | | Move to next question |  | | | |
| Is inheritance filtering used where trios are available? | Yes | | Are exceptions to inheritance filtering included in this pipeline? | Yes | - Predicted deleterious variants (nonsense/frameshift/canonical splice site etc) - Reported in ClinVar/HGMD/Other database as likely pathogenic / pathogenic - Custom Whitelist - Other – please state | Move to next section |  |
|  |  |  |  | No | Move to next section |  |  |
|  | No | | Move to next section |  |  |  |  |
|  | N/A - singleton testing only | | Move to next section |  |  |  |  |
| Variant interpretation - ( single nucleotide variants (SNVs) | | | | | | | |
| Who performs the initial variant interpretation (SNVs)? - tick all that apply | - Laboratory scientist - Clinical Geneticist - Genetic counsellor - Fetal Medicine Clinician - Other – please state | | Move to next question |  |  |  |  |
| Is there a subsequent review of the variant classification (SNVs)? | Yes | | Who performs this secondary review? - tick all that apply | - Laboratory scientist - Clinical Geneticist - Genetic counsellor - Fetal Medicine Clinician - Other – please state | Move to next question |  |  |
|  | No | | Move to next question |  |  |  |  |
| Which guidelines are used for SNV variant interpretation? | - ACMG - UK – ACGS - Other – please state | | Move to next question |  |  |  |  |
| Are SNVs confirmed by a secondary method? | Yes | | - Which secondary method is used to confirm SNVs? | - Sanger sequencing - NGS – using alternative method   Other – please state | Move to next section |  |  |
|  | No | | Move to next section |  |  |  |  |
| Variant interpretation – copy number variants (CNVs) | | | | | | | |
| Are CNVs analysed? | Yes | | What is the limit of CNV detection? | Free type response e.g. Single exon – whole chromosome | Move to next question |  |  |
|  |  |  | Who performs the initial variant interpretation (CNVs)? - tick all that apply | - Laboratory scientist - Clinical Geneticist - Genetic counsellor - Fetal Medicine Clinician - Other – please state | Move to next question |  |  |
|  |  |  | Is there a subsequent review of the variant classification (SNVs)? | Yes | Who performs this secondary review? - tick all that apply | - Laboratory scientist - Clinical Geneticist - Genetic counsellor - Fetal Medicine Clinician - Other – please state | Move to next question |
|  |  |  |  | No | Move to next question |  |  |
|  |  |  | Which guidelines are used for CNV variant interpretation? | - ACMG - UK – ACGS - Other – please state | Move to next question |  |  |
|  |  |  | Are CNVs confirmed by a secondary method? | Yes | Which secondary method is used to confirm SNVs? | Quantitative real time PCR   - Quantitative fluorescent PCR - Digital PCR   Other – please specify | Move to next section |
|  |  |  |  | No | Move to next section |  |  |
|  | No | | Go to next section |  |  |  |  |
| Multidisciplinary team discussions | | | | | | | |
| As part of the service do you have multidisciplinary team discussions? | Yes | | Who is involved in these discussions? - tick all that apply | - Laboratory scientist - Clinical Geneticist - Genetic counsellor - Fetal Medicine Clinician - Other – please state | At what point in the process are discussion held – tick all that apply? | - Reviewing referrals - After variant interpretation prior to reporting - After reporting - Other – please state | Move to next section |
|  | No | | Move to next section |  |  |  |  |
| Reporting | | | | | | | |
| What information is included on the report regarding the result? (tick all that apply) | - Variant using HGVS nomenclature - Whether the variant is consistent with / confirms a diagnosis or not - Criteria used for variant classification - Inheritance of the variant (if trio testing performed) and if prenatal testing should be offered for future pregnancies or offered to other family member at risk - MDT outcome e.g. postnatal phenotypic follow-up recommended - Other – please state | | Move to next question |  |  |  |  |
| What technical information is included on the report? | - Brief description of pipeline, variant prioritisation and classification protocols - Panel version number and link to gene content, if appropriate - Validated sensitivity of pipeline - Mean coverage & contact details on how to find out coverage of specific genes - Other – please state | | Move to next question |  |  |  |  |
| Accreditation | | | | | | | |
| Is the service accredited? | Yes | | To which standard is the service accredited? | - ISO:15819 - ISO:17025 - Other – please specify | Move to next section |  |  |
|  | No | | Move to next section |  |  |  |  |
| External quality assessment | | | | | | | |
| Do you participate in external quality assessment for prenatal exome/genome sequencing? | Yes | | Which schemes do you participate in? | - GenQA - EMQN - Others – please specify | Which areas of prenatal sequencing service are covered by external quality assessment? | - DNA extraction - Library preparation - Sequencing - Bioinformatics pipeline - Variant classification, - Reporting - Other – please state | Move to next section |
|  | No | | Move to next section |  |  |  |  |
| Additional (looked for) findings | | | | | | | |
| Are additional (looked for e.g. cancer susceptibility genes) reported | Yes | | Which genes are incorporated for additional findings? | - ACMG list (provide reference) - Other – please state | Move to next question |  |  |
|  |  |  | Who are additional findings reported in? | - Parents only - Fetus only - All family members | Does your reporting practice differ dependant on if parents or fetus? | Yes (please specify -free text box) | Move to next section |
|  |  |  |  |  |  | No | Move to next section |
|  | No | | Move to next section |  |  |  |  |
| Incidental findings | | | | | | | |
| Are incidental findings reported? (ie variants identified that are not related to the indication for testing but are identified incidentally – NOT looked for as above) | - Yes – always - Yes – if agreed at multidisciplinary team discussion - Other – please state | | Who are incidental findings reported in? | - Parents only - Fetus only - All family members | Move to next section |  |  |
|  | No | | Move to next section |  |  |  |  |
| Reanalysis | | | | | | | |
| Does reanalysis of NGS data happen? | Yes | | When does this reanalysis occur? – tick all that apply | New pregnancy | Move to next section |  |  |
|  |  |  |  | Baby born with new postnatal phenotypic information | Move to next section |  |  |
|  |  |  |  | New version of digital panel | Move to next section |  |  |
|  |  |  |  | New clinical feature develops in childhood | Move to next section |  |  |
|  |  |  |  | Systematic | At what intervals is the data reanalysed? | Free text answer |  |
|  |  |  |  | Other – please state | Move to next section |  |  |
|  | No | | Move to next section |  |  |  |  |
| Data sharing | | | | | | | |
| Do you actively share your variant classification data? | Yes | | Which database do you use – tick all that apply? | - ClinVar - Decipher - Other – please state |  |  |  |
|  | No | | Move to thank you page |  |  |  |  |
